# Attenuation of Retinal Vascular Development in Neonatal Mice Subjected to Hypoxic-Ischemic Encephalopathy

**DOI:** 10.1038/s41598-018-27525-8

**Published:** 2018-06-15

**Authors:** Ismail S. Zaitoun, Ulas Cikla, Dila Zafer, Eshwar Udho, Reem Almomani, Andrew Suscha, Pelin Cengiz, Christine M. Sorenson, Nader Sheibani

**Affiliations:** 1 Department of Ophthalmology and Visual Sciences, University of Wisconsin School of Medicine and Public Health, Madison, WI 53705 USA; 2 McPherson Eye Research Institute, University of Wisconsin School of Medicine and Public Health, Madison, WI 53705 USA; 3 Department of Pediatrics, University of Wisconsin School of Medicine and Public Health, Madison, WI 53705 USA; 4 Department of Neurological Surgery, University of Wisconsin School of Medicine and Public Health, Madison, WI 53705 USA; 5 Department of Biomedical Engineering, University of Wisconsin School of Medicine and Public Health, Madison, WI 53705 USA; 6 Department of Cell and Regenerative Biology, University of Wisconsin School of Medicine and Public Health, Madison, WI 53705 USA

## Abstract

A significant proportion of children that survive hypoxic-ischemic encephalopathy (HIE) develop visual impairment. These visual deficits are generally attributed to injuries that occur in the primary visual cortex and other visual processing systems. Recent studies suggested that neuronal damage might also occur in the retina. An important structure affecting the viability of retinal neurons is the vasculature. However, the effects of HIE on the retinal neurovasculature have not been systemically evaluated. Here we investigated whether exposure of postnatal day 9 (P9) neonatal mice to HIE is sufficient to induce neurovascular damage in the retina. We demonstrate that the blood vessels on the surface of the retina, from mice subjected to HIE, were abnormally enlarged with signs of degeneration. The intermediate and deep vascular layers in these retinas failed to form normally, particularly in the periphery. All the vascular damages observed here were irreversible in nature up to 100 days post HIE. We also observed loss of retinal neurons, together with changes in both astrocytes and Müller cells mainly in the inner retina at the periphery. Collectively, our findings suggest that HIE results in profound alterations in the retinal vasculature, indicating the importance of developing therapeutic strategies to protect neurovascular dysfunction not only in the brain but also in the retina for infants exposed to HIE.

## Introduction

Early visual impairment and childhood blindness can result in significant learning and developmental delays. Childhood blindness increases the socioeconomic burden on the child’s family and society^1,2^. Fortunately, the advancement in the healthcare provided to pregnant mothers and neonates has improved the outcome of neonatal infants that develop retinopathy of prematurity, a major cause of visual impairment in children. However, the same progress in the neonatal and pediatric critical care units paradoxically has also increased the survival rate of neonate and full-term babies with neurological injuries that are frequently associated with visual impairment^3,4^.

Pediatric brain injuries are major causes of visual impairment in developed countries^5–8^. Hypoxic-ischemic encephalopathy (HIE), one of the most common brain injuries, results from secondary oxygen deprivation and blood flow reduction to the brain. Its incidence ranges from 0.1% to 0.8% of live births in developed countries^9^. A considerable proportion of HIE patients display visual impairments^10,11^, which are thought to be associated with lesions in the neural visual structures such as the optic radiations, visual associative cortex area, optic nerve, primary visual cortex, and visual attention pathways^12^. In addition, Nickel and Hoyt^13^ reported that respiratory or cardiac arrests in children were sufficient to cause temporary subnormal electroretinogram. However, whether patients with HIE suffer from local ocular damage is rarely explored.

The Rice-Vannucci model, where permanent unilateral ligation of the common carotid artery (CCA) followed by exposure to a hypoxic air mixture, is one of the most commonly used acute hypoxia-ischemia model in rat^14–16^ and mouse pups^17,18^. In this model, brain damage is limited to the hemisphere ipsilateral to the occluded CCA^19^. The CCA gives rise to the ophthalmic artery, which in turn makes the eye vulnerable to HIE insult. This model is extensively utilized to investigate the impact of HIE on brain development and function. In addition, a few studies have used this model to show the damaging effect of the HIE on either the neuronal component^20,21^ or the vascular component^22^ of the retina in rats. However, to the best of our knowledge, there have not been such investigations conducted in mice, where both the neuronal and the vascular components of the retina are studied.

The development and function of vascular and neuronal systems are intimately linked^23^. Thus, it is important to investigate the effect of HIE on the neurovasculature structure and function. The inner retinal vasculature in mice has three layers: the superficial, deep, and intermediate layers, which develop postnatally in an age-dependent manner^24–26^. The aims of this report were to examine the effects of HIE on the neurovasculature of mouse retina during development, and assess the reversibility of the observed changes post injury.

## Results

### Hypoxic-ischemic encephalopathy disrupts normal vascular development in the mouse retina

We used the mouse model of neonatal HIE by exposing mice for only 50 minutes of hypoxia^15,18,27,28^. Mice with mild to moderate brain injury can suffer from severe retinal damage^21^. Thus, it is important to make the distinction between the brain and the retina when the state of the damage is mentioned. Although this model also results in damage to the brain, we have confined our observations to the retina. Thus, all the damage descriptions presented here in the result and discussion sections apply to the mouse retina.

**Figure 1:**
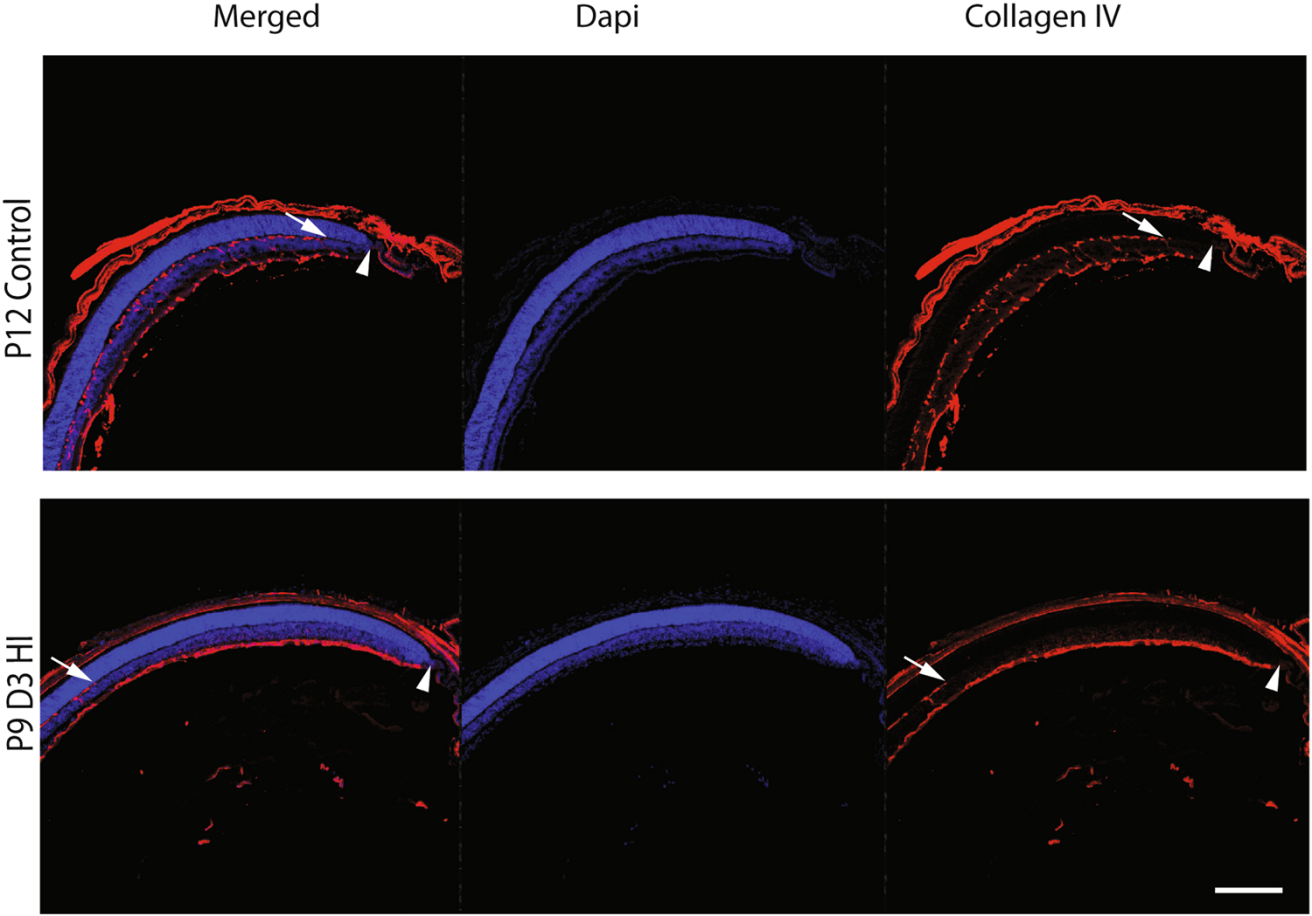
Hypoxic-ischemic insult disrupts the vascular development in the murine neonatal retina. Representative photomicrographs were taken from the middle to the periphery of cryosectioned P12 control and P9D3 HIE retinas. Blood vessels were labeled with Collagen IV and all retinal nuclei were labeled with DAPI. Collagen IV staining (red) demonstrate an approximate complete coverage of the deep vascular layer (arrow) to the periphery of the P12 control retinas (arrow head) but not of the P9D3 HIE retinas. Eyes from three mice per group were examined. Scale bar, 20 µm.

HIE in the rat neonate causes vascular degeneration of targeted areas of the brain^29,30^. To assess whether exposing neonatal mice to HIE has any damaging effect on the normal postnatal development of the retinal vasculature, we first examined eye cryosections taken from P9D3 HIE (3 days post-HIE) and P12 control mice (Fig. 1). The inner retinal vasculature in mice has three layers: the superficial, deep, and intermediate layers that develop during the first three weeks of postnatal life^24–26^.

Eye sections were immunostained for collagen IV, a vascular basement membrane protein to label blood vessels, and DAPI to label nuclei in the retina. As compared to retinas from P12 control mice, the vascular development in the retina from P9D3 HIE mice was abnormal particularly in the mid-periphery of the retina (Fig. 1). In the retinas from P9D3 HIE mice, the deep vascular layer and the perpendicular vessels sprouting from the superficial layer were absent. In addition, blood vessels of the superficial layer appeared abnormally dense and thick.

**Figure 2:**
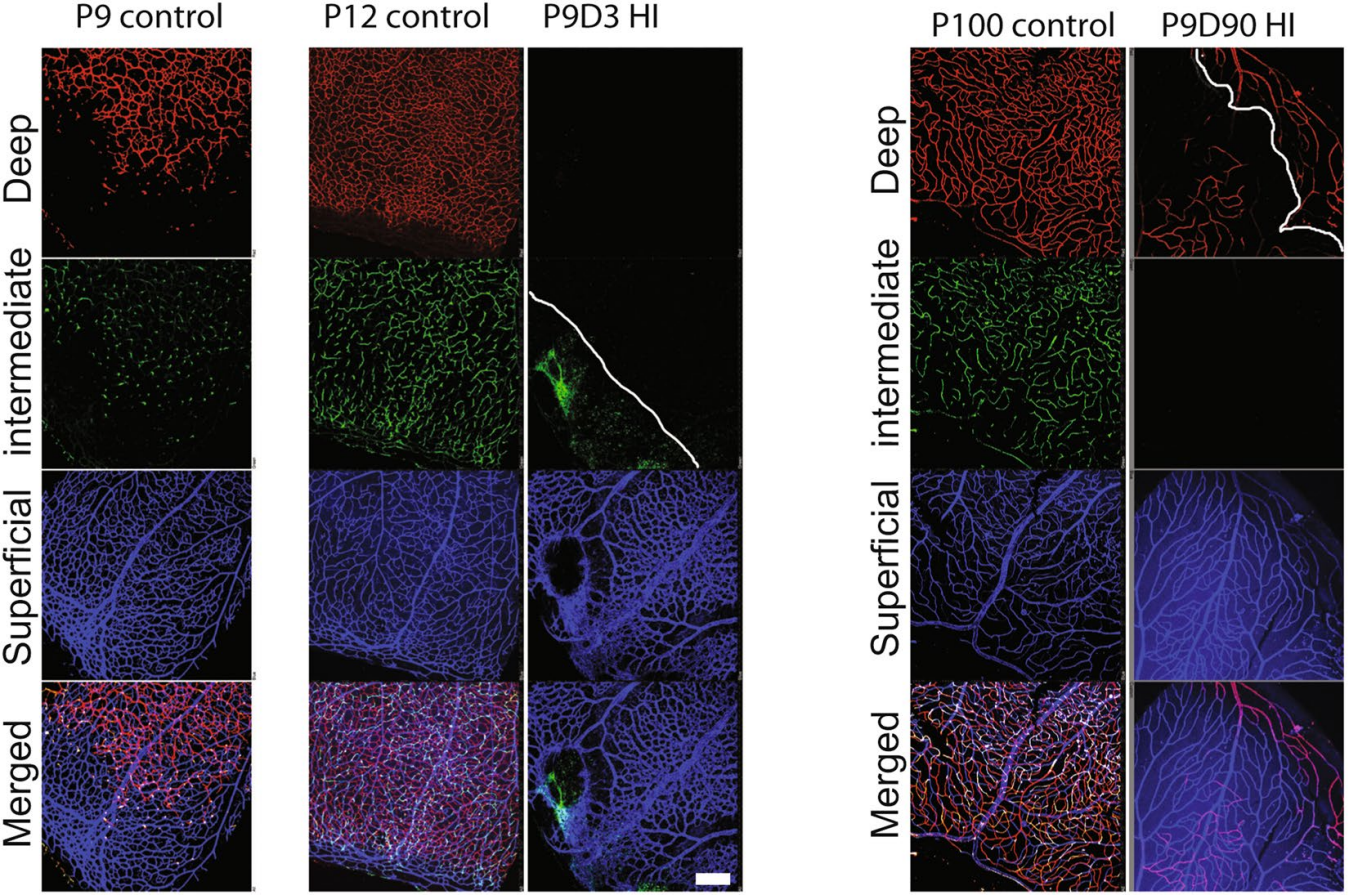
Hypoxic-ischemic insult disrupts the vascular development in the murine neonatal retina. Representative photomicrographs from the middle to the periphery of the retinas were taken from P9 control, P12 control, P9D3 HIE, P100 control, and P9D90 HIE mice. Blood vessels were labeled with Collagen IV or Griffonia simplicifolia isolectin B4. Vessels in different retinal layers were given pseudo-colors (Red, deep vascular layer; Green, intermediate vascular layer; blue, superficial layer). Because of retinal thinning, the superficial vascular layer at the edge of periphery is at the same plane of the lagging deep vascular layer; Both layers are separated by the white solid line. Retinas from at least five mice per group were examined. Scale bar, 50 µm.

To better characterize the effects of HIE on the developing retinal vasculature, we immunostained wholemount retinas for collagen IV, which were imaged using confocal microscopy. Figure 2 shows representative retinal vascular profiles from P9, P12, and P100 control, and P9D3 and P9D90 HIE mice. A retinal vascular profile from a P9 control mouse is included to represent the stage of retinal vascular development prior to HIE. At P9, the superficial layer has reached the periphery, while the deep layer is still advancing peripherally to cover the remaining one-third of the retina. The intermediate layer has only perpendicular sprouting vessels, corresponding to the advancement of the deep vascular layer. At P12 in control mice, the superficial layer was most mature, the deep and the intermediate layers were advanced but still developing- a profile typical of vascular development in the P12 C57BL/6J mouse retina^24–26^. In contrast, retinal vascular structure in P9D3 HIE mice was severely disrupted. The superficial vascular layer was disorganized and had signs of endothelial hyperplasia as the vessels were thick and dense; the deep and intermediate layers were completely absent from the retinal periphery. The retinal vasculature from P9D90 HIE mice demonstrated that the vascular damage becomes more obvious with age and the retinal thinning was evident at this age. Using the confocal z-stack acquisition and maximum intensity projection, we imaged and separated the three vascular layers of retinas taken from P9D3 HI, P12 control and P100 control mice. Contrary to other groups, a smaller number of z-stack images were sufficient to go through the full thickness of the retinas taken from P9D90 HIE mice. In addition, using the maximum intensity projection it was difficult to display the deep vascular layer alone, especially at the periphery of the retina. Please note the appearance of the peripheral vasculature of the superficial layer in the deep vascular plane above the solid white line in the image (Fig. 2).

**Figure 3:**
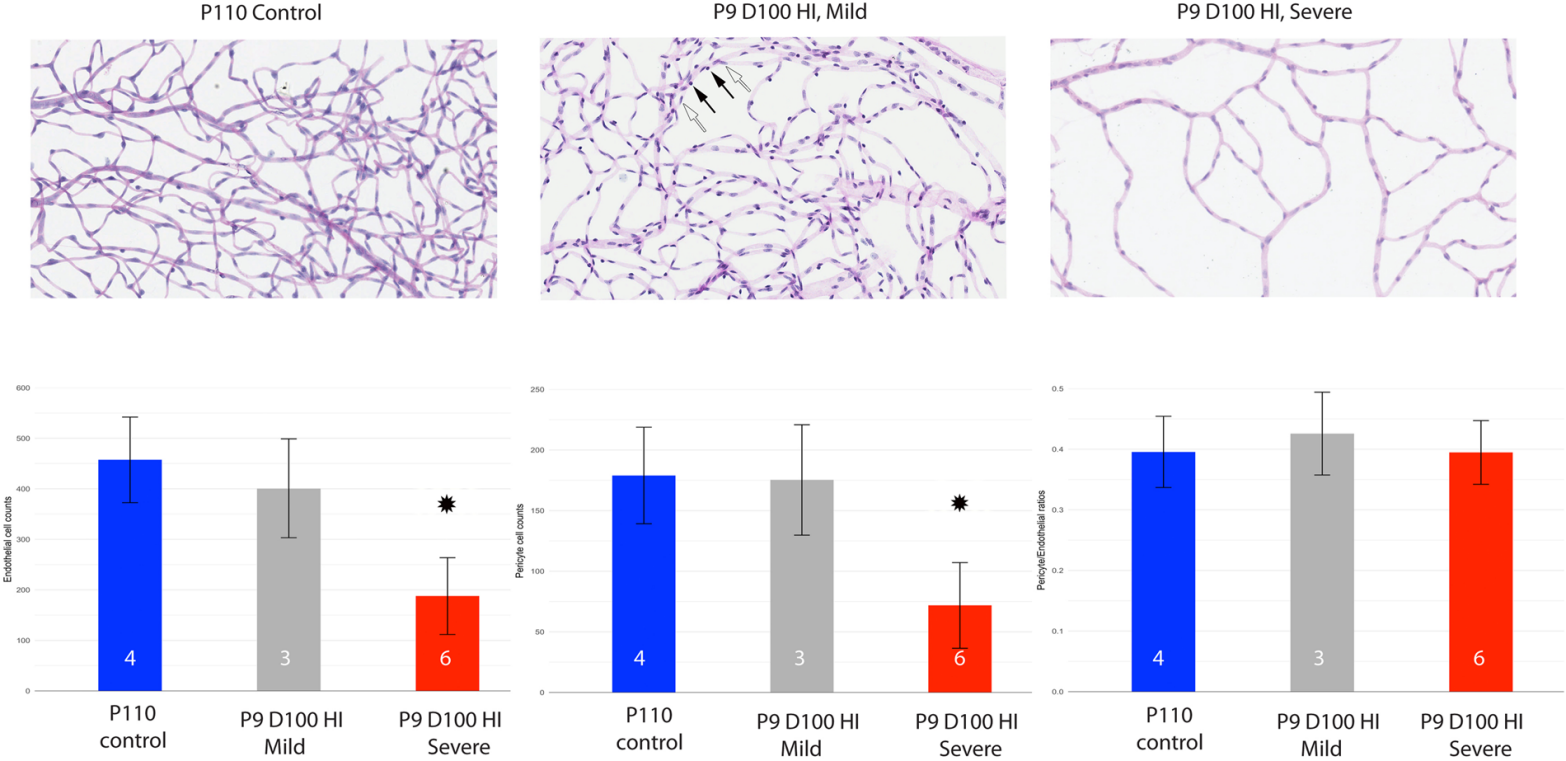
Hypoxic-ischemic insult disrupts the vascular development in the murine neonatal retina as shown by trypsin digest preparations. Representative photomicrographs were taken from the peripheral retinal vasculature of P110 control, P9D100 mildly injured HIE and P9D100 severely injured HIE retinas. The nuclei of endothelial cells lie within the vessel wall along the axis of the capillary (empty arrow), while pericyte nuclei generally have a protuberant position on the capillary wall (solid arrow). Retinas from four control, 3 mild and 6 severe HIE mice were examined (numbers shown in the bars). The bars show the estimated mean cell counts/ratios. The error bars are 95% confidence intervals. Both means and CIs were estimated using the linear mixed effects models. * Indicates that by our criteria the HIE severe group is significantly different from control group. Images were captured on Aperio slide scanner and x20 objective was used.

To better assess whether endothelial cell and pericyte numbers in the developing retinal vasculature were affected by HIE, we prepared retinal trypsin digests from control and mice subjected to HIE. Visual inspection of the vascular preparations from HIE mice allowed for easy separation of these retinas according to the severity of injury, either as mild (33%; 3 out of 9 HIE retinas) or severe (67%; 6 out of 9 HIE retinas) groups. The mild P9D100 HIE group appeared similar to the control group, with very little noticeable vascular loss in the retinal periphery. In contrast, the severe group demonstrated an obvious global low retinal vascular density (Fig. 3; Figure S1). Noteworthy, all HIE data shown throughout this document, except Figs 3 and S1, represent the severe form of retinal neurovascular damage.

To quantify the damage, we determined the number of endothelial cells and pericytes as well as the pericyte to endothelial ratio (PC/EC) from the retinal periphery of trypsin digest preparations (Fig. 3). The numbers of endothelial cells and pericytes were similar for the mildly injured and the control groups. However, the group with severe injury had significantly lower endothelial cell and pericyte numbers compared with their age-matched controls. The PC/EC ratios were not significantly different from the control group, suggesting that HIE resulted in a proportional decrease in the numbers of both retinal endothelial cells and pericytes. To assess whether loss of endothelial cell and pericytes was due to capillary degeneration, we counted the number of acellular capillaries in the trypsin digest preparations. Interestingly, we found no significant differences in the number of acellular capillaries among the HIE and control groups (not shown).

### Hypoxic-ischemic encephalopathy results in the formation of neovascular tufts similar to those observed in retinas from mice subjected to oxygen-induced ischemic retinopathy

**Figure 4:**
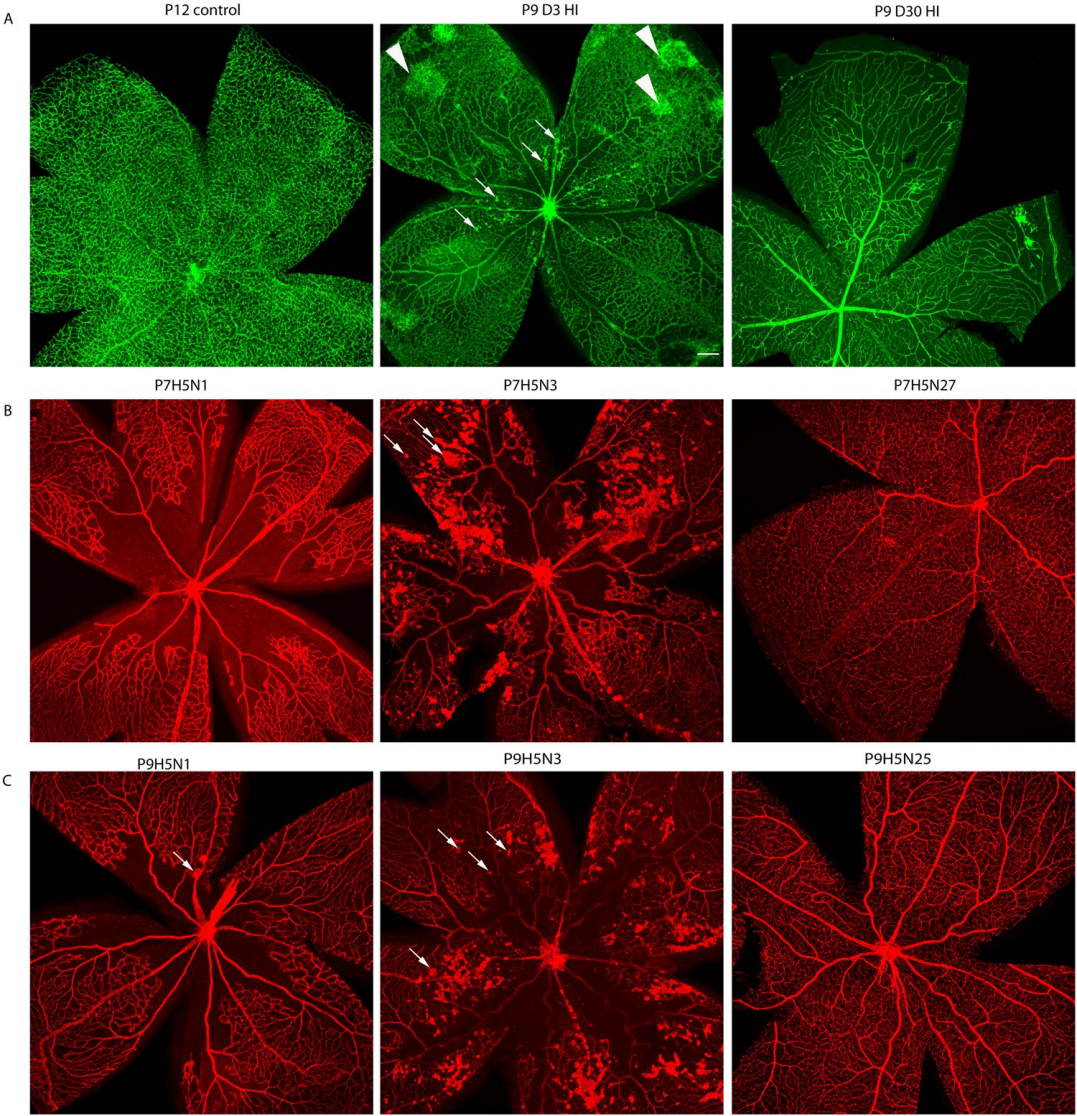
Hypoxic-ischemic insult occasionally results in the formation of neovascularization tufts. Representative photomicrographs were taken from P12 control, P9D3 HIE, and P9D30 HIE retinas (**A**), P7H5N1, P7H5N3, and P7H5N27 retinas and (**B**), P9H5N1, P9H5N3, and P9H5N25 retinas (**C**). Blood vessels were labeled with Griffonia simplicifolia isolectin B4 or collagen IV. Neovascular tufts are marked by arrows. Arrowheads indicate unique abnormal vascular structures that do not protrude toward the vitreous. Retinas from at least four mice per time-point were examined. Scale bar, 200 µm.

The retinas from P9D3 HIE mice occasionally developed sparse and individual neovascular tufts in the central retina, especially on major veins and capillaries (Fig. 4). The tufts could be seen 2 days after the HIE (P9D2 HIE), the earliest time-point investigated after the onset of HIE. These neovascular tufts were not observed on arteries. Neovascular tufts were observed in retinas that suffered from a dramatic absence of blood vessels in the intermediate and deep retinal layers, specifically in the middle-periphery towards the center of the retina (Fig. 4). Nevertheless, no neovascular tufts were observed in retinas of P9D30 or P9D90 HIE mice. Interestingly, the HIE-induced neovascularization we observed was similar to that noted in mice subjected to oxygen-induced ischemic retinopathy (OIR).

Intrigued by this similarity, we next compared HIE-induced vascular damage described in this report to the vascular damage that occurs in the mouse retina during OIR. During OIR, P7 mice are subjected to a hyperoxic environment (75% oxygen) for 5 days (P7H5), and then returned to room air (normoxia; 20% oxygen) for 5 days (P7H5N5). We performed this assay on P7 mice and their retinas were analyzed at P7H5N0 (age-matched to P9D3 HIE mice), P7H5N1, P7H5N2, P7H5N3, P7H5N4, P7H5N5 and P7H5N27 (age-matched to P9D30 HIE mice). Figure 4 shows representative images from P7H5N1, P7H5N3, and P7H5N27 retinas. All blood vessels in the three layers in the center of the retina were absent during the hyperoxic exposure, with the exception of the main arteries and veins in the superficial layer. The vascular void in the center of the retina becomes hypoxic-ischemic upon return of the animals to room air. To overcome this unique type of hypoxic-ischemic condition induced by hyperoxia, new blood vessels start developing in two forms. The pathologic neovascularization form, gives rise to vascular tufts that grow into the vitreous. Neovascular tufts were first seen at P7H5N2 and peaked in P7H5N5 mice with a considerable number of vascular sheet-like structures, a sign of aggressive neovascularization. The neovascular tufts were observed on the remaining veins, capillaries and to a lesser extent on arteries throughout the retina but were concentrated at the zone surrounding the obliterated area.

The second type of newly formed blood vessels arises from physiological vascularization, a condition that typically results in revascularization of the whole avascular area of the retina. Retinas taken from P7H5N27 mice were fully vascularized in the three vascular layers (Fig. 4). This was concomitant with regression of the majority of neovascular tuffs. All OIR phenotypes observed here are similar to other previous reports^31^. To be comparable to the age of the HIE insult model, which started with P9 mice, the OIR model was next performed by exposing P9, instead of P7, mice to hyperoxia. Figure 4 shows representative images taken from retinas analyzed at P9H5N1, P9H5N3, P9H5N25 time points. Interestingly, the area of vascular obliteration did form in the center of retinas from P9H5N1 mice subjected to hyperoxia, albeit a smaller area when compared with that of P7H5N1 retinas. A few neovascular tufts started to form as early as P9H5N1 and resolved by P9H5N5, in contrast with retinas from P7H5N5 mice. In addition, retinas from P9H5N5 mice showed a fully vascularized superficial layer which did not extend to the deep and intermediate layers. Retinas from P9H5N25 mice showed a fully vascularized three layers of vasculature (Fig. 4). Using the confocal z-stack acquisition, the three vascular layers of retinas taken from OIR mice were easily imaged and separated (not shown), suggesting that the neuroretina of these mice did not show considerable neuronal loss, an observation reported by others as well^32^.

### Hypoxic-ischemic encephalopathy results in retinal gliosis

**Figure 5:**
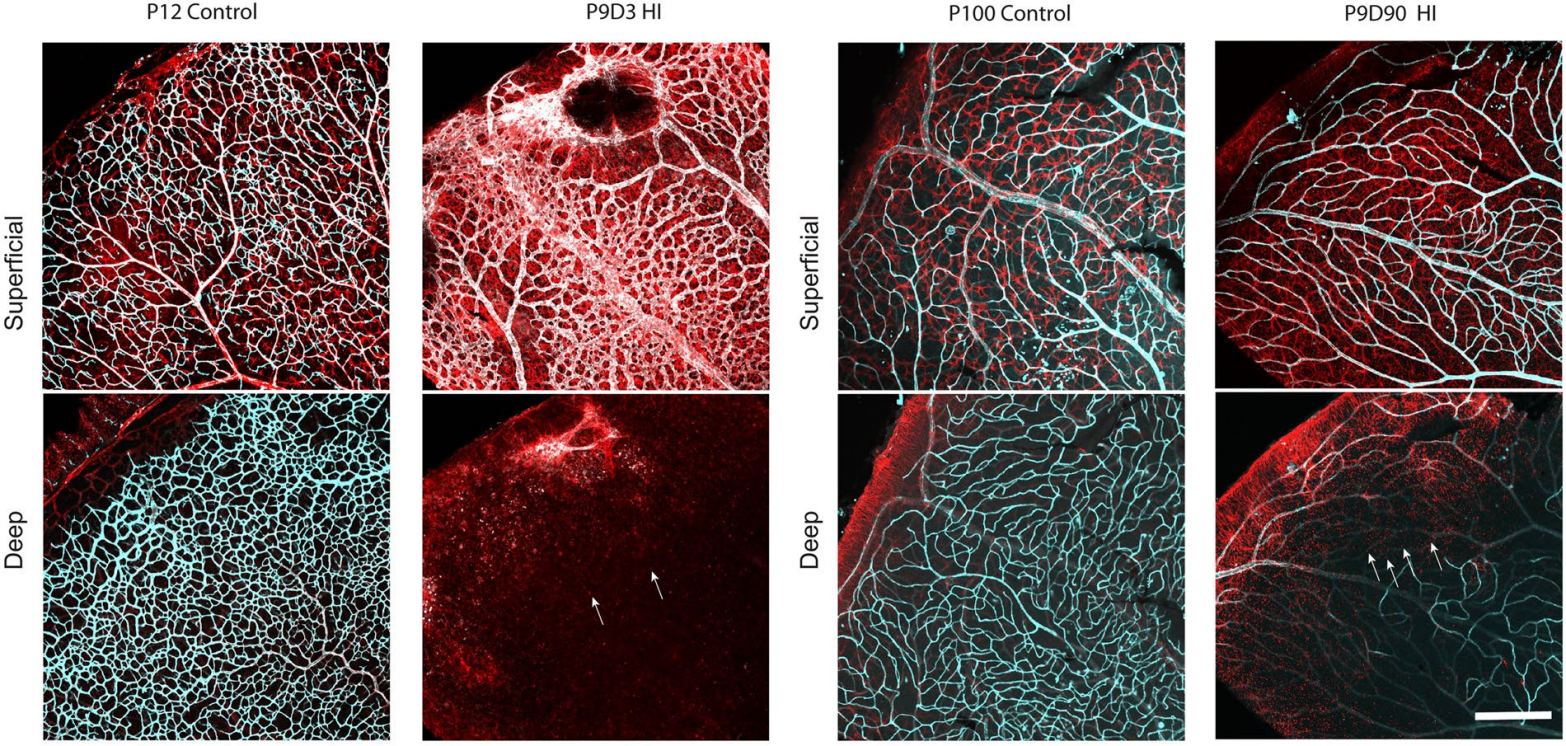
Astrocyte distribution and density were altered in the retinas of HI mice. Representative photomicrographs were taken from P12 and P100 control and P9D3 and P9D90 HIE mice. Flat-mount retinas were labeled for isolectin B4 and GFAP, an intermediate filament protein expressed in astrocytes an in activated Müller cells. Two separate maximum projection images were generated per representative sample at the superficial and deep vascular layers. Retinas from control animals showed typical astrocyte distribution and density (stellate-shaped astrocytes which makes discrete contacts on blood vessels)^35^. On the other hand, the majority of HI retinas showed altered astrocytes distribution and density and activation of Müller cells. Arrows represent the activated Müller cells. Retinas from at least five mice per group were examined. Scale bar, 200 µm.

Upregulation of the intermediate filament proteins, including glial fibrillary acidic protein (GFAP) and vimentin, is the hallmark of glial activation^33,34^. To evaluate the distribution and the activation status of astrocytes in the retina following HIE, retinas from control and HIE mice were stained as wholemounts with an anti-GFAP antibody. Figure 5 illustrates representative images taken from retinas of P12 and P100 controls and P9D3 and P9D90 HIE mice. In retinas from both control and HIE mice, astrocytes were present in the ganglion cell and nerve fiber layer. Morphologically, astrocytes in the retinas from control mice of either age showed a stellate appearance, where the soma is compacted and easily recognizable and the processes are thin. Also, retinas from control mice showed a typical astrocyte distribution, where their cell bodies are interacting but without forming bundles or scar-like structures^35^.

In contrast, astrocytes in the retinas from P9D3 HIE mice were activated and lost their stellate appearance. The processes appeared thicker, bundled, and randomly connected to the soma. Furthermore, retinas from P9D3 HIE mice frequently showed scar-like structures especially at the periphery of the retina. The spatial-specific astrogliosis is consistent with the neuronal damage that occurs more frequently at the periphery of the retina. In P9D90 mice, retinal astrocytes tended to regain their stellate shape and lose their hypertrophic appearance. However, they stayed densely populated compared to age-matched controls. Müller cells remained active in the retinas from P9D90 mice, as demonstrated by the sand-like GFAP positive staining, which was not observed in retinas from P12 or P100 control mice (Fig. 5). Figure S2 shows low magnification images taken from P12, P39 and P100 control and P9D3, P9D30 and P9D90 HIE mice. Astrogliossis can be easily seen in nearly all retinas from HIE mice. Careful visual assessment of retinas for GFAP staining showed that on average 77% [100% of P9D3 (10 out of 10), 60% of P9D30 (6 out of 10), and 66% (4 out of 6) of P9D90] of retinas from HIE animals displayed an obvious astrogliosis, especially at the periphery. In contrast, astrogliosis was completely absent in retinas from control animals.

### Hypoxic-ischemic encephalopathy induces damage in the developing mouse neuroretina

**Figure 6:**
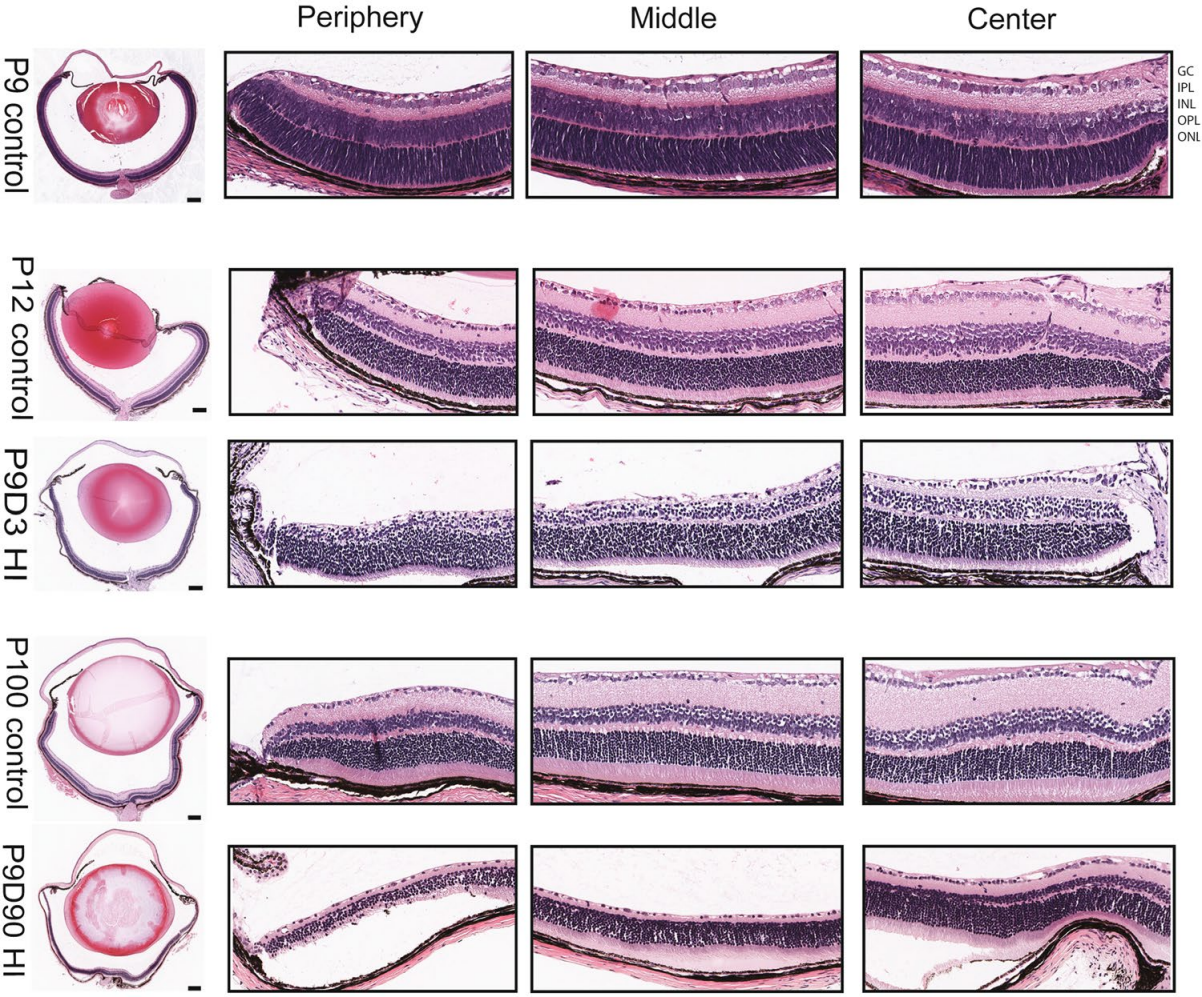
Hypoxic-ischemic insult causes degeneration of murine neonatal neuroretina. Eyes were processed and paraffin-sectioned through the optic disc then hematoxylin and eosin stained. Representative photomicrographs were taken for the whole eye section and from the center, the middle and the periphery of the eye. Postnatal day 9 (P9) control eye represents the eye structure before the hypoxia-ischemia was initiated; P12 control eye; P9D3 HIE represent P12 mouse (3 days post-HI); P100 control eye; P9D90 represent P99 mouse (90 days post-HI). Abbreviations: GCL: ganglion cell layer; IPL: inner plexiform layer; INL: inner nuclear layer; OPL: outer plexiform layer; and ONL: outer nuclear layer. Images were captured on Aperio slide scanner. Eyes from three mice per group were examined. Scale bar, 200 µm.

To evaluate the integrity of retinal morphology of mice exposed to HIE, histological sections were prepared from animals at three-time points: i) postnatal day 9 (P9), HIE start time; ii) P9D3, three days after HIE start time; and iii) P9D90, ninety days after HIE start time. Eyes from naïve mice of the same age were used as controls. Surprisingly, exposing animals to 50 min of hypoxic exposure caused ocular damage ranging from mild to severe. Representative retinal images from the center, middle and periphery of eyes from naïve and mice with severe ocular damage are shown in Fig. 6. Visual inspection of the retinal sections taken from P9, P12 and P100 naïve mice showed gradual thinning of the retinal nuclear layers and gradual thickening of the plexiform layers, a normal developmental process which reflects the maturation status of the retinal neurons^36^. Three days after HIE, damage was observed in all retinal layers. The inner nuclear layer and the inner and outer plexiform layers were most affected, and the outer nuclear layer was least affected. Ninety days after HIE, all layers continued to degenerate such that the outer plexiform layer disappeared and the inner and outer nuclear layers merged resulting in fewer cell layers. The damage appeared to vary in a gradient across the retina eccentricity: periphery of the insulted mice was most damaged, and the center was least damaged.

## Discussion

Here we studied the effect of neonatal HIE on retinal neurovascular integrity. We observed a profound irreversible disruption and delay in the normal retinal vascular development of approximately two thirds of the mice subjected to HIE. These changes were concomitant with irreversible loss of the inner neuroretina that occurred at the periphery. These results are clinically relevant to surviving term neonatal HIE patients with visual impairment.

Our studies suggest that HIE caused irreversible damage to the retinal deep and intermediate vascular layers, especially at the periphery. The superficial vascular layer was less damaged, similar to HIE effect on rat retinal vasculature^22^. In line with our findings in the retina, other HIE studies of the neonatal animal brain demonstrated severe and permanent vascular loss in the targeted hemisphere^37,38^. In addition, others have reported that focal arterial stroke in neonatal animals results in irreversible degeneration of the already existing vasculature and attenuates further angiogenic activity^39^. The developmental stage at which HIE takes place determines the pattern and severity of degeneration of the central nervous system. Seminal work by Licht *et al*.^40^ demonstrated that the vascular maturation status dictates the susceptibility of neurons in the neonatal brain to HIE^40^. Perhaps this also dictates the susceptibility of the retina to HIE, as our studies suggest.

We observed occasional neovascular protrusions pointing toward the vitreous, a pattern similar to the neovascular tufts typically observed in the retina of mice subjected to OIR. The neovascular tufts occur in HIE retinas with severe deep and intermediate vascular damage could be caused by the upregulation of pro-angiogenic growth factors, such as vascular endothelial growth factor (VEGF) by the neuroretina. ROP is a major cause of visual impairment in humans worldwide. We investigated the vascular responses in the mouse retina during OIR where hyperoxia exposure started at either P7 or P9. Retinas from both animal groups developed the typical pathologic phenotypes, which then later became fully vascularized concomitant with regression of neovascular tufts, as previously reported by others^31,32^. Interestingly, successful retinal revascularization in mice suffering from hyperoxia-induced retinopathy suggests that the cellular and/or molecular cues critical for angiogenesis are relatively preserved or regained with time. In striking contrast, HIE appeared to induce an irreversible damage to the retinal vasculature such that the cellular and/or molecular cues critical for angiogenesis were not preserved. The reasons for differences in the ability of the retina to respond to ischemia-mediated angiogenesis in mice subjected to HIE compared with OIR are not known, but may be linked to irreversible loss of inner retinal neurons following HIE.

Reactive gliosis is a common retinal response to stress. Under chronic conditions, gliosis may intensify neurodegeneration by compromising the integrity of the blood retinal barrier, thereby, allowing the possible accumulation of toxic materials^41,42^. Although astrocytes showed less GFAP immunoreactivity with time, alterations in their morphology and distribution persisted even three months following HIE insult. Furthermore, retinal Müller cell gliosis increased with time. Thus, the reactive gliosis we observed in our studies likely contributes to the neuronal and vascular damage as well as the attenuation of revascularization in the retinas of mice subjected to HIE.

HIE induced retinal neuronal damage especially in the inner retina (GCL, IPL, INL, and OPL). The retinal periphery was more severely affected compared with the center. These observations are similar to the finding of other groups where rat pups were exposed to 2 hours of hypoxic insult. Huang *et al*.^20^ exposed P7 rat pups to HI insult and found that the inner retinal structure was specifically damaged while the outer retina was relatively spared. Similar findings were reported when P10 rat pups were exposed to HI insult^21^. Although both studies found no direct relationship between functional or structural damage in the brain compared with the retina, they do suggest that the retinal damage is highly likely to play a role, at least in part, in the visual impairments associated with HIE. The neuroretina damage induced following HI insult may be caused by accumulation of free oxygen radicals, glutamate excitotoxicity, inflammation and compromised vascular barrier function^43^. However, the contribution of these pathways to irreversible loss of retinal inner neurons and suppression of ischemia mediated neovascularization following HIE needs further investigation.

The superficial vascular layer in the retina starts to form after birth (P0) by radially spreading off the angiogenic sprouts from near the optic disc towards the periphery, which is completed at P8. The deep vascular layer is second to develop after the superficial layer. It forms when perpendicular vascular sprouts start to come out of veins, venules and capillaries closer to the optic nerve area at P7. They move towards the OPL border, and then spread radially to form the deep vascular layer, which is completed by P11-P12. The intermediate layer, the last to form, is completed by ~P15. The primary retinal vascular plexus is interconnected and completed by P21^25,26^. The formation of superficial retinal vascular layer is controlled by ganglion cells and astrocytes^44–46^. The formation of deep vascular layer is controlled by Müller cells and horizontal cells^24,47,48^, while the intermediate retinal vascular layer is controlled by amacrine cells^47^. Thus, the spatial-temporal developmental pattern of the three retinal vascular layers is intertwined with the development of different neurons in the retina: Retinal ganglion cells are the first type of neurons to be generated, and horizontal cells and cones are second to be generated. Then amacrine cells are generated which are followed by the last to form cell types: Müller glia, bipolar cells and rods^36^. Furthermore, the spatial-temporal birth gradient, from the center towards the periphery of the retina, for any specific type of neuron could overlap with the birth of other types of neurons. The retinal vasculature develops to supply nutrients and oxygen to the growing and maturing retina. This suggests that the three retinal vascular layers receive their potentially distinct angiogenic and vascular homeostatic signals, for the most part, from the neurons and glial cells that they are built to support. This may explain the order of the formation of the three vascular layers in the retina under physiologic conditions. The irreversible and severe neuronal and vascular damage we observed here is consistent with the notion that immature cells are very vulnerable to HI insults. However, whether the immature vasculature in the retina is responsible for the retinal neurons injuries, as in the brain^40^, is unknown and requires further investigation.

The distinct vascular and neuronal degeneration we observed as a result of the HI is similar to phenotypes observed in retinas of mouse models where von Hippel–Lindau tumor suppressor (*Vhl)*^49–53^, Hypoxia-inducible factor-1α (*Hif1α*)^53^, or Hypoxia-inducible factor-2α (*Hif2α*)^54^ genes were lacking. Of relevance to our data, conditional deletion of *Vhl* and *Hif1α*, specifically in amacrine and horizontal cells, disrupted the normal vascular development in the intermediate and deep vascular layers^47^. Taken together, the similarity of our observations to the phenotypes of the *Vhl*, *Hif1α* and *Hif2α* knockout mice suggests that mild and acute HI insult may cause permanent functional disruption of the Vhl, Hif1α, Hif2α or some of their targeted genes.

It is important to note that isoflurane was used during the HIE procedure, which potentially could have masking effects to some of the phenotypes shown in this report. Isoflurane has neuroprotective effects in neonatal hypoxic-Ischemic brain injury^55^. Another limitation of this study is that Fig. 6 represents a small number of samples. However, the data presented herein is in line with previous reports^20,21^, which showed that the inner layers of the retina were permanently damaged. Also, the level of the retinal damage was assessed visually based on the integrity of the vasculature in the three layers of the retina. However, the quantitative assessments of trypsin digest preparations demonstrate significant changes in retinal vascular density.

In conclusion, we show that the integrity of the retinal neurovasculature is severely compromised in mice following HIE. This suggests that neonatal infants with HIE should be carefully followed for neurovascular dysfunction not only in the brain but also in the retina.

## Materials and Methods

### Ethics Statement and animals

Experiments were performed in accordance with the National Institutes of Health Guide for the Care and Use of Laboratory Animals and approved by the Institutional Animal Care and Use Committee of the University of Wisconsin School of Medicine and Public Health. C57BL/6J mice were obtained from the Jackson Laboratory. Mice were housed in a sterile environment and were allowed *ad libitum* access to standard rodent chow and water. The day of the birth was considered postnatal day zero (P0). Animals were euthanatized using either isoflurane or carbon dioxide.

### Induction of term neonatal HIE

Hypoxia and ischemia were induced in postnatal day nine (P9) C57BL/6J mice. P9 pups are commonly used in neonatal hypoxic-ischemic studies conducted on mice. Naïve C57BL/6J mice were used as controls. The pups were anesthetized with isoflurane (Butler Schein Animal Health Supply, Reno, NV) (5% for induction, 2–3% for maintenance) in 30% oxygen mixed with nitrous oxide. The body temperature of the pups were maintained at 36 °C using a heated surgical table (Molecular Imaging Products, Bend, OR). Under a surgical microscope (Nikon SMZ-800 Zoom Stereo, Nikon, Melville, NY), a midline skin incision was made and the trachea was visualized through the muscle overlying it. The left common carotid artery was freed from the left common jugular vein and vagus nerve by blunt dissection, electrically cauterized and cut. In addition to the brain, the hypoxic-ischemic insults generated by this procedure apply to the eye as well; the ophthalmic artery is a branch from the common carotid artery. The incision was injected with 0.5% bupivacaine and closed with a single 6.0 silk suture. Animals were returned to their dams and monitored continuously for a 2 h recovery period. To induce unilateral ischemic injury, the animals were placed in a hypoxia chamber (BioSpherix Ltd, Redfield, NY) equilibrated with 10% O_2_ and 90% N_2_ at 36 °C for 50 min. This is a well-characterized model of neonatal HIE and results in reproducible brain injury ipsilateral to the electrocauterized left common carotid artery 15, 18, 27, 28.

### Oxygen-induced ischemic retinopathy

Oxygen-induced ischemic retinopathy (OIR) was induced in C57BL/6J mice by exposing either P7 or P9 pups to 75 ± 0.5% oxygen in an air-tight incubator for a period of 5 days. The temperature of the incubator was maintained at 23 ± 2 °C, and oxygen was continuously monitored with a PROOX model 110 oxygen controller (Reming Bioinstruments Co., Redfield, NY). At the end of the 5-day period, animals were gradually introduced to room air over a period of 5–6 hours before they were transferred into their regular housing area. Animals were kept under room air conditions until desired time-points were reached. Naïve C57BL/6J mice were used as controls.

### Histology

Eyes were enucleated and submerged in 10% formalin at 4 °C for overnight. Eyes were then washed three times in phosphate buffered saline (PBS) placement in 70% ethanol at 4 °C for overnight and processed for embedding in paraffin. Thin tissue sections (5 μm) were prepared through the optic disc and the center of cornea, and allowed to adhere to glass slides. Eye sections were stained with hematoxylin and eosin. The sections were imaged using an inverted microscope (Olympus TH4-200; Olympus, Tokyo, Japan), which is equipped with a digital camera (Olympus U-TV0.63xc). Nikon NIS-Elements AR 4.50.00 software was used to assess the integrity of the eye.

### Staining frozen sections

Eyes were enucleated and embedded in optimal cutting temperature (OCT) compound and 10 µm sections were prepared. Sections were fixed in 4% paraformaldehyde (PFA) on ice for 20 min, washed with PBS, blocked for one hour (3% protease-free BSA and 0.3% Triton X–100 in PBS) at room temperature. The primary antibodies were prepared in blocking solution and added to the sections and kept rocking for 3 h at room temperature. Slides were then washed three times in PBS before applying the secondary antibodies. Both primary and secondary antibodies were prepared as indicated in the wholemount immunostaining (see below). Eye sections were viewed by fluorescence microscopy and images were captured in digital format using either an Evos microscope or Nikon confocal microscope system A1+.

### Immunofluorescence staining of wholemount retinas

Eyes were enucleated and fixed in 4% PFA for 10 min at room temperature, washed three time in PBS, and then transferred to 70% methanol and kept at −20 °C until stained. The day of staining, eyeballs were rehydrated in PBS for one hour on a rocker at room temperature. Retinas were dissected in PBS and then washed in PBS three times, 10 min each, and incubated in the blocking solution [3% protease-free bovine serum albumin (BSA), and 0.3% Triton X-100 in PBS] for one hour. Retinas were then incubated with primary antibodies diluted (1/250) in the blocking solution at 4 °C overnight. Following incubation, retinas were washed three times with PBS, 10 min each, incubated with fluorescently conjugated secondary antibodies, diluted (1/500), for 2 h at room temperature (RT), washed four times with PBS, 30 min each, and mounted on a slide with DAPI Fluoromount-G (Southern Biotech). Retinas were viewed by fluorescence microscopy and images were captured in digital format using Nikon confocal microscope system A1+. Captured images were analyzed using NIS elements viewer (Nikon). Primary antibodies used: rabbit anti-collagen IV (Millipore, AB756P), Goat anti-collagen IV (Southern Biotech, 1340-01), Rat anti-pecam1-Alexa 488 (AbD Serotec, MAC2388A488), Goat anti-pecam1 ((R&D Systems, AF3628), mouse anti-GFAP-Alexa555 (Cell Signaling, 3656S), and rabbit anti-GFAP (Cell signaling, 12389S). Biotinylated Griffonia Simplicifolia Lectin I (GSL I) isolectin B4 (Vector Laboratories, B-1205) was also used to stain the vasculature. All secondary antibodies and conjugated streptavidin were obtained from Jackson ImmunoResearch Laboratories including donkey anti-rabbit-Cy2 (cat. No. 711-225-152), donkey anti-rabbit-Cy3 (cat. No. 711-165-152), donkey anti-rabbit-Cy5 (cat. No. 711-175-152), donkey anti-goat-Cy3 (cat. No. 705-165-147), donkey anti-goat-Cy5 (cat. No. 705-175-147), streptavidin-alexa fluor 488 (ThermoFisher; cat. No. 016-540-084), streptavidin-alexa fluor 647 (ThermoFisher; cat. No. 016-600-084) and streptavidin-cy3 (Jackson ImmunoResearch; cat. No. 016-160-084). Occasionally, images were pseudo colored to enhance visual presentation.

### Trypsin-digested retinal vessel preparations

Animals were euthanatized using high dose of either isoflurane or carbon dioxide. Eyes were enucleated and submerged in 4% PFA for at least one week. The cornea, lens and the choroid were dissected out and the whole retina was retrieved under the dissecting microscope. Retinas were washed three time in PBS, rinsed twice in distilled water and then washed in fresh distilled water overnight at room temperature. The next morning, distilled water was decanted and the retinas incubated in 3% trypsin (Trypsin 1:250, Difco) prepared in 0.1 M Tris, 0.1 M maleic acid, pH 7.8 containing 0.2 M NaF for approximately 12–16 h at 37 °C overnight. The following morning, retina was retrieved and nonvascular cells were loosened and separated away from the vasculature. Clean retinal vessels were radially cut proximal to the optic nerve four times and then flat-mounted on glass slides for periodic acid-Schiff (PAS) and hematoxylin staining. Nuclear morphology was used to distinguish pericytes from endothelial cells. The nuclei of endothelial cells are oval or elongated and lie within the vessel wall along the axis of the capillary, while pericyte nuclei are small, spherical, stain densely, and generally have a protuberant position on the capillary wall. The stained and intact retinal wholemounts were coded, and subsequent counting was performed. One sampling image (0.273 square mm, each) was taken from periphery of each of the four quadrants of the retina. Thus, endothelial cells and pericytes in an area of 0.273 × 4 = 1.09 square mm were counted per retina. Care was taken to avoid counting from areas where the vasculature folded on itself on the slide (please see Figure S1). Stained nuclei of endothelial cells and pericytes were used to distinguish and determine their numbers per field of view of retinal vasculature. Only retinal capillaries were included in the cell counts, which were performed in the periphery of the retina. We counted the number of endothelial cells and pericytes in four fields of view from the four quadrants of each retina. To evaluate the density of cells in the capillaries, the mean number of endothelial cells or pericytes was recorded in four fields of view from the four quadrants of each retina. To assess acellular capillaries, trypsin-digested vessels of the retina were prepared as described above. Acellular capillaries were quantified in the same field areas used to count the endothelial cells and the pericytes. Acellular capillaries were recognized as collapsed capillary-sized tubes with no nuclei along their whole length.

### Grading the retinal damage

Visual inspection of the retinal vasculature was used to determine the levels of the damage, which were classified into mild, moderate and severe forms. Damage was considered mild when the deep vascular layer was only absent in the retinal periphery while the rest of the retina has intact intermediate and superficial vascular layers. On the other hand, damage was considered moderate when the deep vascular layer was absent from the whole retina while the rest of the retina has intact intermediate and superficial vascular layers. Damage was considered severe when the deep vascular layer was absent from the periphery and the middle (the area between the periphery and the center) of the retina and the intermediate and superficial vascular layers throughout the whole retina were also damaged.

### Statistical Analysis

Based on visual assessment of the trypsin digest preparations, three separate groups were considered: P110 control, mild P9D90 HIE, and severe P9D90 HIE groups. As an exploratory analysis of the data, visual inspection and descriptive statistical analysis were done. For all linear mixed effects models, the residuals were checked for normality using QQ-plots. In all cases, the normality assumption was not violated. Data from mild P9D90 HIE and severe P9D90 HIE were independently compared to data from P110 control group. Linear Mixed Effects models were fitted to the data to explore the effect of the severity on the cell count. A random effect of sample ID was included. Estimated mean counts with 95% confidence intervals were calculated by a bootstrap method based on 100,000 simulations. Intervals with no overlap were considered statistically different. The means presented in the figures are estimated using the Linear Mixed Effects models that include sample ID as a random effect.

The analyses were performed using the lme4 package^56^ in the statistical software R.

## Electronic supplementary material

Supplementary information
