# Supplementary material for: Attenuation of Retinal Vascular Development in Neonatal Mice Subjected to Hypoxic-Ischemic Encephalopathy
Source: Sci Rep. 2018 Jun 15;8:9166. doi: 10.1038/s41598-018-27525-8 (PMC6003906; doi:10.1038/s41598-018-27525-8)

Supplementary Information

Attenuation of Retinal Vascular Development in Neonatal Mice Subjected to Hypoxic-Ischemic Encephalopathy

Ismail S. Zaitoun[1,2*], Ulas Cikla[3, 4], Dila Zafer[3], Eshwar Udho[3], Reem Almomani[1], Andrew Suscha[1], Pelin Cengiz[3], Christine M. Sorenson[2,3], Nader Sheibani[1,2,5,6]

Department of Ophthalmology and Visual Sciences[1], McPherson Eye Research Institute[2], Department of Pediatrics[3], Department of Neurological Surgery[4], Department of Biomedical Engineering[5], and Department of Cell and Regenerative Biology[6], University of Wisconsin School of Medicine and Public Health, Madison, WI 53705

*Corresponding Author:
Ismail Zaitoun, PhD
University of Wisconsin
Department of Ophthalmology and Visual Sciences
1111 Highland Avenue, WIMR 9418
Madison, WI 53705
iszaitoun@wisc.edu

Figure S1. Hypoxic-ischemic insult disrupts the vascular development in the murine neonatal retina as shown by trypsin digest preparations. Representative photomicrographs were taken from the whole retinal vasculature of P110 control, P9D100 Mildly injured HIE and P9D100 severly injured HIE retinas. Three representative preparations from each group are shown. Images were captured on Aperioslide scanner and x20 objective was used.

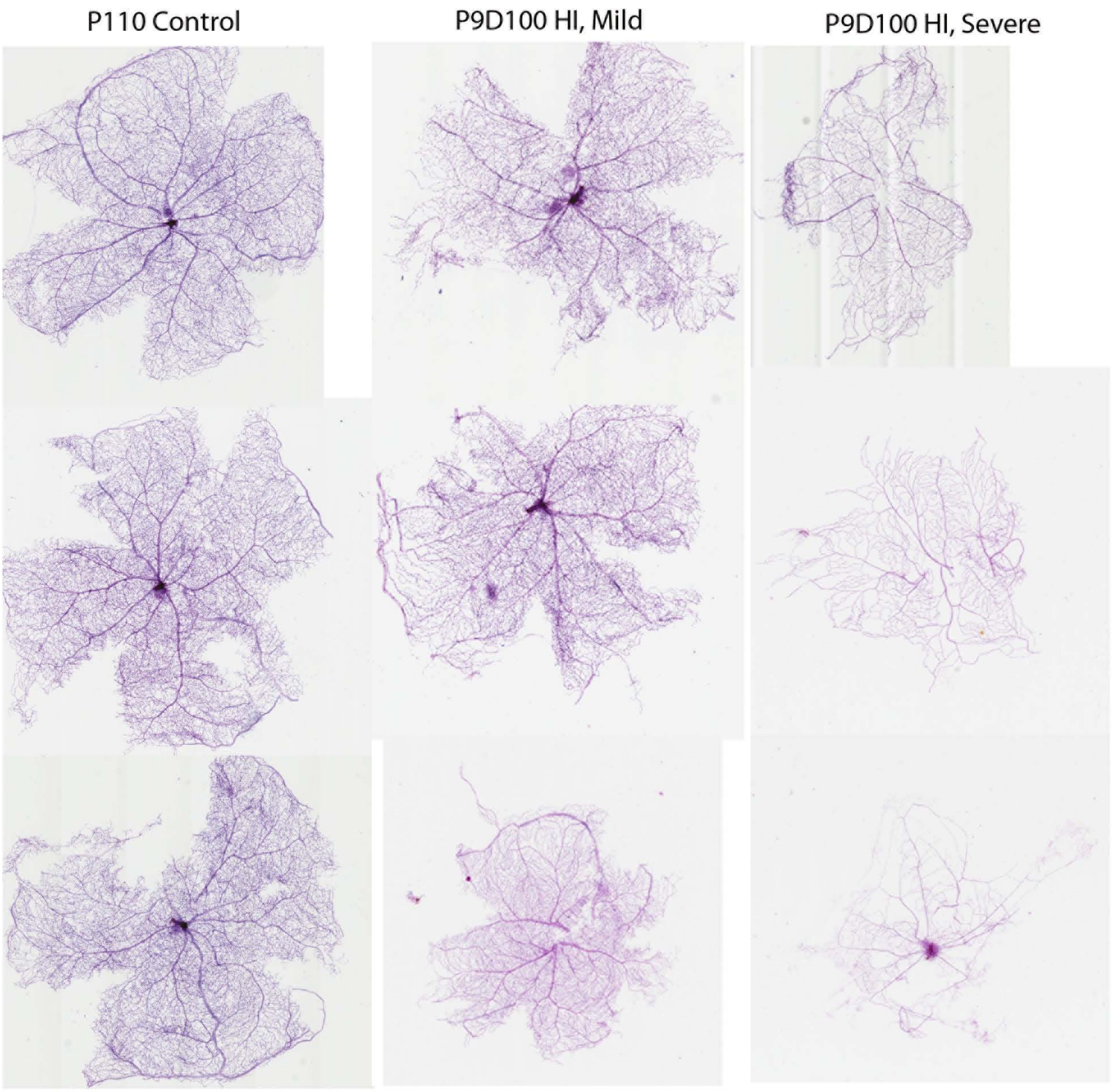

Figure S2. Astrocyte distribution and density was altered in the retinas of HI mice. Representative photomicrographs were taken from P12, P39 and P100 control and P9D3, P9D30 and P9D90 HIE mice. Flat-mount retinas were labeled for GFAP. Retinas from control animals showed typical astrocyte distribution and density, while the majority of HIE retinas showed altered astrocytes distribution and density and activation of Müller cell, as shown by the intense GFAP staining (dashed lines). Scale bar, 1000 µm.

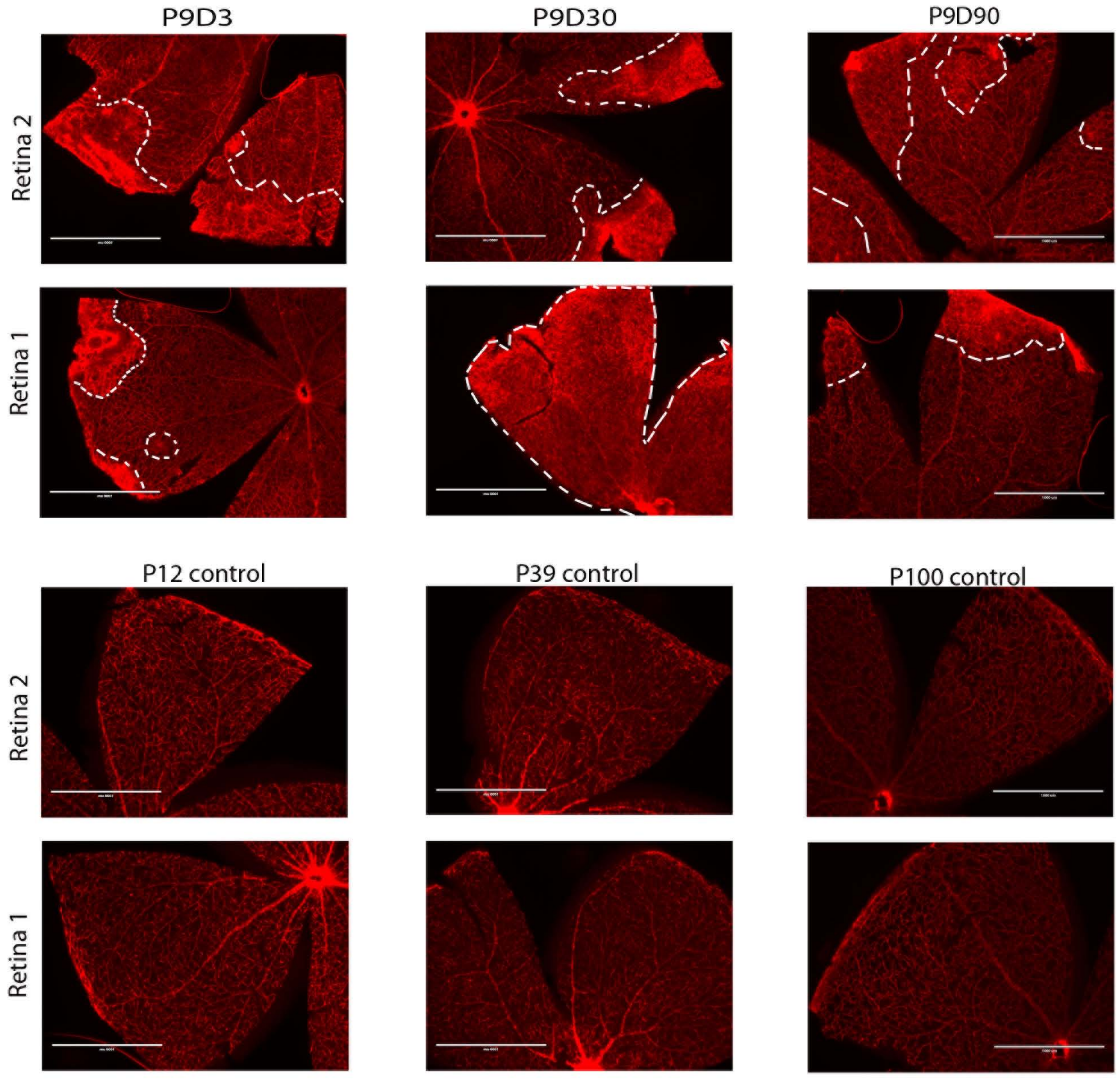

Supplement: Supplementary file 1 — Supplementary information [file 41598_2018_27525_MOESM1_ESM.pdf]
